# Spike count and morphology in the classification of epileptiform discharges

**DOI:** 10.3389/fneur.2023.1165592

**Published:** 2023-05-23

**Authors:** Eivind Aanestad, Nils Erik Gilhus, Henning Kristian Olberg, Mustafa Aykut Kural, Sándor Beniczky, Jan Brogger

**Affiliations:** ^1^Department of Clinical Neurophysiology, Haukeland University Hospital, Bergen, Norway; ^2^Department of Clinical Medicine, University of Bergen, Bergen, Norway; ^3^Department of Neurology, Haukeland University Hospital, Bergen, Norway; ^4^Department of Clinical Neurophysiology, Danish Epilepsy Centre Filadelfia, Dianalund, Denmark; ^5^Department of Clinical Neurophysiology, Aarhus University Hospital, Aarhus, Denmark; ^6^Department of Clinical Medicine, Aarhus University, Aarhus, Denmark

**Keywords:** epileptiform, morphology, count, quantitative, EEG, validation

## Abstract

**Purpose:**

The purpose of this study is to investigate the impact of Bergen Epileptiform Morphology Score (BEMS) and interictal epileptiform discharge (IED) candidate count in EEG classification.

**Methods:**

We included 400 consecutive patients from a clinical SCORE EEG database during 2013–2017 who had focal sharp discharges in their EEG, but no previous diagnosis of epilepsy. Three blinded EEG readers marked all IED candidates. BEMS and IED candidate counts were combined to classify EEGs as epileptiform or non-epileptiform. Diagnostic performance was assessed and then validated in an external dataset.

**Results:**

Interictal epileptiform discharge (IED) candidate count and BEMS were moderately correlated. The optimal criteria to classify an EEG as epileptiform were either one spike at BEMS > = 58, two at > = 47, or seven at > = 36. These criteria had almost perfect inter-rater reliability (Gwet’s AC1 0.96), reasonable sensitivity of 56–64%, and high specificity of 98–99%. The sensitivity was 27–37%, and the specificity was 93–97% for a follow-up diagnosis of epilepsy. In the external dataset, the sensitivity for an epileptiform EEG was 60–70%, and the specificity was 90–93%.

**Conclusion:**

Quantified EEG spike morphology (BEMS) and IED candidate count can be combined to classify an EEG as epileptiform with high reliability but with lower sensitivity than regular visual EEG review.

## Introduction

The definition of epileptiform activity includes qualitative criteria to guide EEG readers in detecting interictal epileptiform discharges (IEDs) (1). According to the criteria, typical morphological traits of an IED are a spiky peak, a wave duration that is different from the background waves, an asymmetric waveform, a slow after-wave, disrupted background activity, and a dipole suggesting that the source of the transient is in the brain. The diagnostic value of morphological IED features has been discussed extensively (2–5). Inter-rater agreement (IRA) has been assessed for specific morphological features (2, 4), and optimal combinations of the criteria have been validated (3).

The IED criteria describe the evaluation of single transients without discussing the possible role of recurring sharp transients. It is unclear whether additional EEG phenomena such as IED counts are relevant for a diagnosis of epilepsy. An EEG conclusion should be based not only on a single graphoelement but also on all available data in an EEG recording. IRA seems to be better for overall EEG interpretation than individual IEDs (4, 6–9). The literature is sparse regarding spike count in routine scalp EEG. Kural et al. (10) demonstrated that a higher IED count is required to conclude that an EEG contains epileptiform activity when the IEDs have a less typical epileptiform morphology. Spike count has been assessed for specific epilepsy types, such as continuous spike–wave during sleep (11, 12), benign epilepsy with centro-temporal spikes (13), temporal lobe epilepsy (14), and juvenile myoclonic epilepsy (15). Latency to the first IED, a measure analogous to spike count has been examined in long-term EEG recordings (16).

We have previously described the Bergen Epileptiform Morphology Score (BEMS) (17), a score from 0 to 86 for sharp transients, where a higher value indicates a more typical epileptiform morphology. BEMS is calculated from carefully selected and visually relatable morphological IED features (spike slope, spike amplitude, spike similarity to background, and slow after-wave area) and patients’ age, as IED morphology depends on age (18). BEMS classified the first sharp discharge in an EEG with a similar performance as an EEG rater both regarding the EEG conclusion (AUC = 0.86) and a future epilepsy diagnosis (AUC = 0.70). With BEMS as an established score for the single sharp discharge, it is possible to assess additional factors that may be relevant to spike identification. This study aimed to examine whether the combination of BEMS and the number of sharp discharges, referred to as IED candidate count in this study, can improve the diagnostic performance when classifying EEGs as epileptiform or non-epileptiform.

## Materials and methods

### Patients and EEGs

We selected a random subsample of EEGs from the total material described in our previous study (17). The original material included all consecutive patients who had standard EEGs or sleep-deprived EEGs recorded in our EEG laboratory at Haukeland University Hospital during the period of 4 March 2013–29 October 2017, which were reported in SCORE EEG (19). We included only those patients who had all their EEGs recorded at our laboratory during the inclusion period, no epileptiform activity in prior EEGs (interictal epileptiform activity or non-focal IEDs), and no prior clinical diagnosis of epilepsy (ICD-10 G40/G41 since 1999) in their hospital medical records. The first EEG for each patient that contained an epileptiform or non-epileptiform sharp discharge was analyzed. Hospital database records were examined for a clinical epilepsy diagnosis until 27 November 2019. The subsample of EEGs selected for this study was then randomized again and divided into two equally sized datasets (DS1 and DS2). DS1 was reserved as a training set to find optimal cut points for the predictor variables. An external dataset (DS3), described by the study mentioned in (10), was used for external validation. In total, 30 out of 60 patients in DS3 had epilepsy, confirmed by recording of their habitual paroxysmal events during long-term EEG monitoring (LTM). The patients included in DS3 were 1 year or older with a median age of 33 years. Their 20-min interictal EEG had to contain sharp transients. Patients were excluded if their LTM was inconclusive regarding epileptic or non-epileptic seizures.

### EEG recordings

Electrodes were applied according to the 10–20 system, with a minimum of 21 and a maximum of 26 electrodes. The 26 electrode montages included three subtemporal electrodes on each side and Fpz. The recording length was 20 min for standard EEGs and 60 min for sleep-deprived EEGs. The sampling rate was 500 samples per second. NicoletOne™ EEG system was used to record and display EEGs for the clinical EEG classification which was used as an outcome, while EEGs were displayed in EEGLAB (20) for the marking of IED candidates.

### IED candidates

We defined IED candidates as sharp transients that could be suspected to be IED, excluding physiological transients (21) and artifacts that mimic epileptiform discharges. Three clinical neurophysiologists, with at least 6 years of experience in EEG interpretation, marked all IED candidates chronologically in each EEG until a maximum count of 40, using a tool that has been described previously (17, 18). Only the channel in which the IED candidate had the most typical epileptiform features was marked for quantitative morphological analysis. We defined a maximum count to reduce the workload and with the assumption that higher counts would not have a significant impact on the performance of classification. In the event of spike trains or IEDs in close temporal proximity, only one distinct spiky component was analyzed per epoch of 1 s. If no IED candidate was identified in an EEG, a negative peak from the background activity on the last page of the EEG recording was marked instead. The IED candidates were marked independently by the three raters (rater 1–3) and blinded to patient data, any previous EEG markings, and the ordinary clinical EEG report. Raters 1 and 2 marked IED candidates in DS1, DS2, and DS3, while rater 3 marked in DS2 and DS3 to increase the number of raters for validation.

### IED candidate-derived diagnostic markers

The following diagnostic markers were derived from the marked IED candidates in DS1:

- BEMS_max_: The IED candidate with the highest BEMS in an EEG.
- BEMS_sum_: The sum of BEMS for all IED candidates in one EEG.
- IED candidate count: The number of IED candidates in one EEG.
- Diagnostic classifier: We searched through combinations of three pairwise IED candidate counts and BEMS thresholds to find the combination with the highest average diagnostic accuracy and IRA when applied as three criteria-sets, where one criteria-set had to be fulfilled to classify an EEG as epileptiform. All combinations were assessed for raters 1 and 2 in DS1 with pre-specified constraints for computational feasibility as follows: The range of BEMS was constrained between 40 and 70 points for the first criteria-set, 30 and 60 points for the second criteria-set, and 20 and 50 points for the third criteria-set. In addition, the BEMS threshold differences between criteria-sets could not be less than 10 points. A total of 5,456 combinations were assessed. The number of criteria-sets was chosen based on the combination of criteria sets given in the study by Kural et al. (10). Adding further criteria-sets was considered to be too computationally demanding, with diminishing returns regarding diagnostic performance.

In addition to the diagnostic markers, the mean BEMS for all IED candidates in one EEG, defined as BEMS_mean_, was calculated.

### Statistics

The diagnostic markers BEMS_max_, BEMS_sum_, IED candidate count, and the binary classification by the diagnostic classifier were grouped according to the EEG conclusion (focal IED or sharp transient). As a secondary outcome measure, these markers were grouped according to whether the patients were diagnosed with epilepsy or not during the follow-up. The diagnostic performance was assessed by measures of sensitivity, specificity, accuracy (the percentage that was correctly classified), and IRA. We calculated the intraclass correlation coefficient (ICC) as a measure of IRA between the raters. IRA between raters for binary classifications was calculated as Gwet’s AC1 (22). Pearson’s correlation coefficient was calculated for BEMS and IED candidate counts. Optimal cut points for the diagnostic markers were chosen in DS1 for raters 1 and 2 as the lowest possible value that corresponded to the highest accuracy, with specificity of >90% for the EEG conclusion. When the optimal cut points differed between the raters, the mean defined the common cut point, except for the diagnostic classifier, where a joint set of combinations was selected that maximized the sum of accuracy and Cohen’s kappa for raters 1 and 2. The performance of the diagnostic classifier and the diagnostic markers was finally assessed in DS2 and DS3, regarding the EEG-conclusion of non-epileptiform transients and IED and the diagnosis of epilepsy during the follow-up. Since candidate counts were limited between 1 and 40, we calculated the estimated candidate count with a Poisson model with 1 as the lower censoring limit and 40 as the upper censoring limit.

## Results

### Patient and EEG characteristics

The original material from our previous study contained a total of 14,337 EEGs. A total of 4,473 EEGs were excluded due to an incomplete EEG history in the SCORE database, a previous diagnosis of epilepsy, or missing data. In total, 6,607 EEGs were excluded because no IEDs or sharp transients had been scored in the clinical report, or the EEG contained a seizure. A subsample of 400 EEGs from 400 different patients was randomly selected from the remaining 2,063 candidates and divided equally into DS1 and DS2. A total of 383 patients were analyzed after excluding 17 patients due to technical difficulties in the process of loading or reading the EEG. Patient age distributions were similar in DS1 and DS2, with a mean age of 39 years and standard deviation (SD) of 28 years (Table 1). In total, 42 out of 383 patients (11%) died during follow-up. The proportion that had EEGs containing clinically scored IEDs differed between DS1 (21%) and DS2 (13%), while those diagnosed with epilepsy were 30% in DS1 and 27% in DS2. The IED candidate count had a wide range with a maximum of 40/min. The estimated mean candidate peak rate was only 0.1–0.4/min for the three raters, which corresponds to 2–8 suspicious peaks in a 20-min EEG.

**Table 1:** Patient and EEG characteristics.

	**DS1**			**DS2**			**Total**		
Sample size, *n*=	196			187			383		
Age in years, mean (SD) (min-max)	37.3	(28.3)	(0–100)	40.5	(26.7)	(0–94)	38.9	(27.5)	(0–100)
Death rate during follow-up, %	12.8			9.1			11.0		
EEG-conclusion IED, %	21.4			13.4			17.5		
Epilepsy at follow-up, %	30.1			27.3			28.7		
IED candidates per minute for rater 1 (Censored), mean (min-max)	1.4	(0.0–25.6)		1.1	(0.0–23.2)		1.2	(0.0–25.6)	
IED candidates per minute for rater 1 (Uncensored), mean	0.36			0.34			0.35		
IED candidates per minute for rater 2 (Censored), mean (min-max)	1.3	(0.0–39.6)		0.6	(0.0–16.8)		1.0	(0.0–39.6)	
IED candidates per minute for rater 2 (Uncensored), mean	0.12			0.09			0.10		
IED candidates per minute for rater 3 (Censored), mean (min-max)	*			1.0	(0.0–22.9)		1.0	(0.0–22.9)	
IED candidates per minute for rater 3 (Uncensored), mean	*			0.14			0.14		

*: Rater 3 did not mark IED candidates in DS1.

### Relationship between IED candidate count and spike morphology

**Figure 1:**
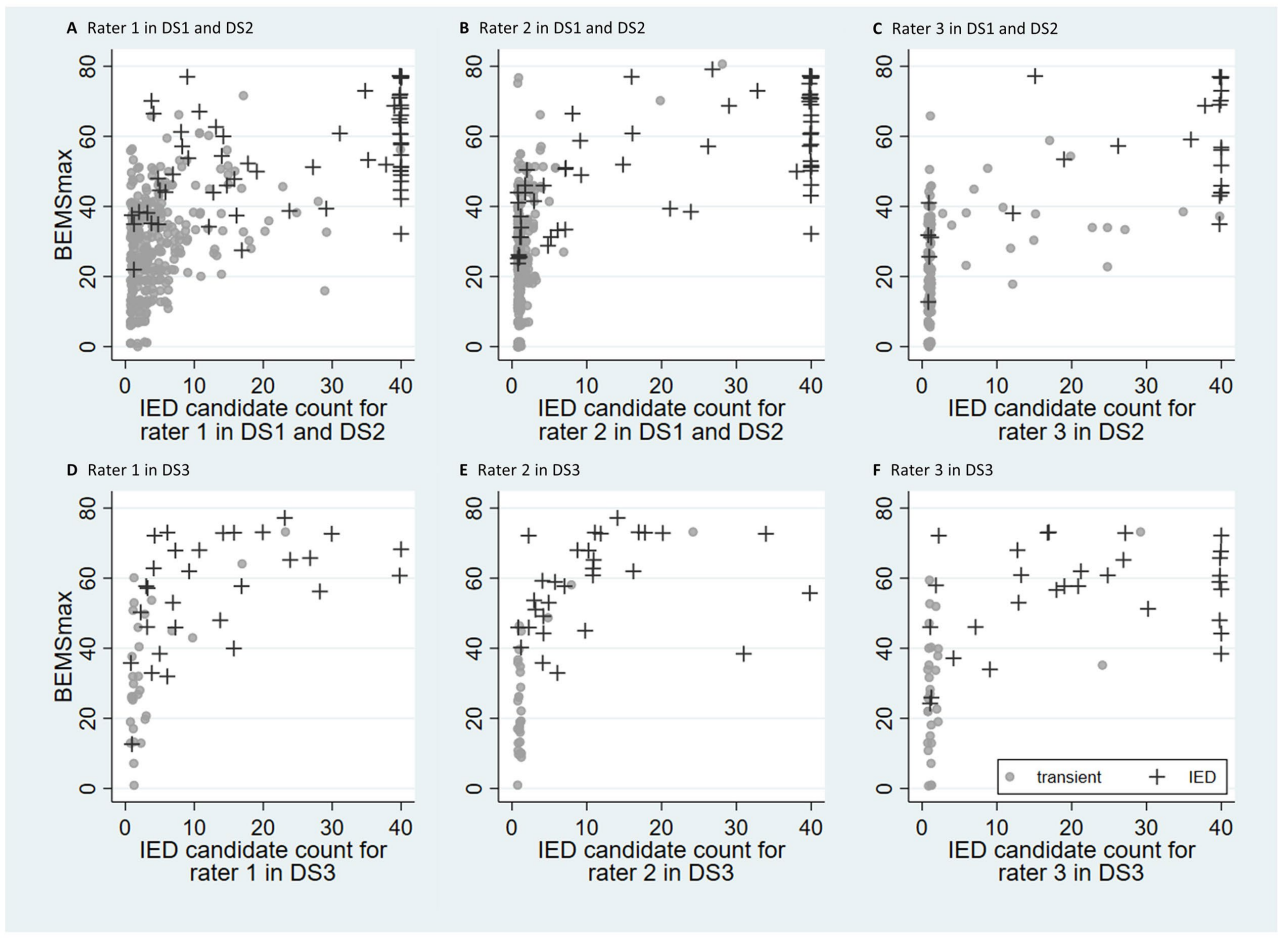
Scatter plots with IED candidate count and BEMS_max_ for the three raters. The datasets DS1 and DS2 (*n* = 383) are shown in **(A–C)** (rater 3 examined DS2 only). The dataset DS3 (*n* = 60) is shown in **(D–F)**. Observations are labeled according to epileptiform (+) or non-epileptiform (●) EEG in DS1 and DS2, and epilepsy (+) or not epilepsy (●) in DS3. Jitter (0.4) has been added to increase visibility of overlapping symbols. Most EEGs in DS1 and DS2 **(A–C)** fell into two clusters, similar for all three raters. The cluster in the lower left corner contains EEGs with infrequent IED candidates and a low BEMS_max_, while the cluster in the upper right corner contains EEGs with frequent IED candidates with a high BEMS_max_. A minority of the EEGs were scattered between the two clusters. This two-cluster pattern was less evident for DS3 **(D–F)**.

Spike morphology and IED candidate count had a positive association in each of the three datasets (Figure 1). BEMS_max_ and IED candidate count had a correlation coefficient (CC) of 0.62 for rater 1, 0.67 for rater 2, and 0.66 for rater 3 in DS1 and DS2 combined (DS2 only for rater 3). The CC for raters 1, 2, and 3 in DS3 was 0.62, 0.60, and 0.61, respectively. BEMS_mean_ and IED candidate count had a correlation of 0.41, 0.42, and 0.37 in the combined DS1 and DS2 for raters 1, 2 and 3, respectively. The significance level was *p* < 0.001 for all correlation coefficients.

### Diagnostic performance in DS1

**Figure 2:**
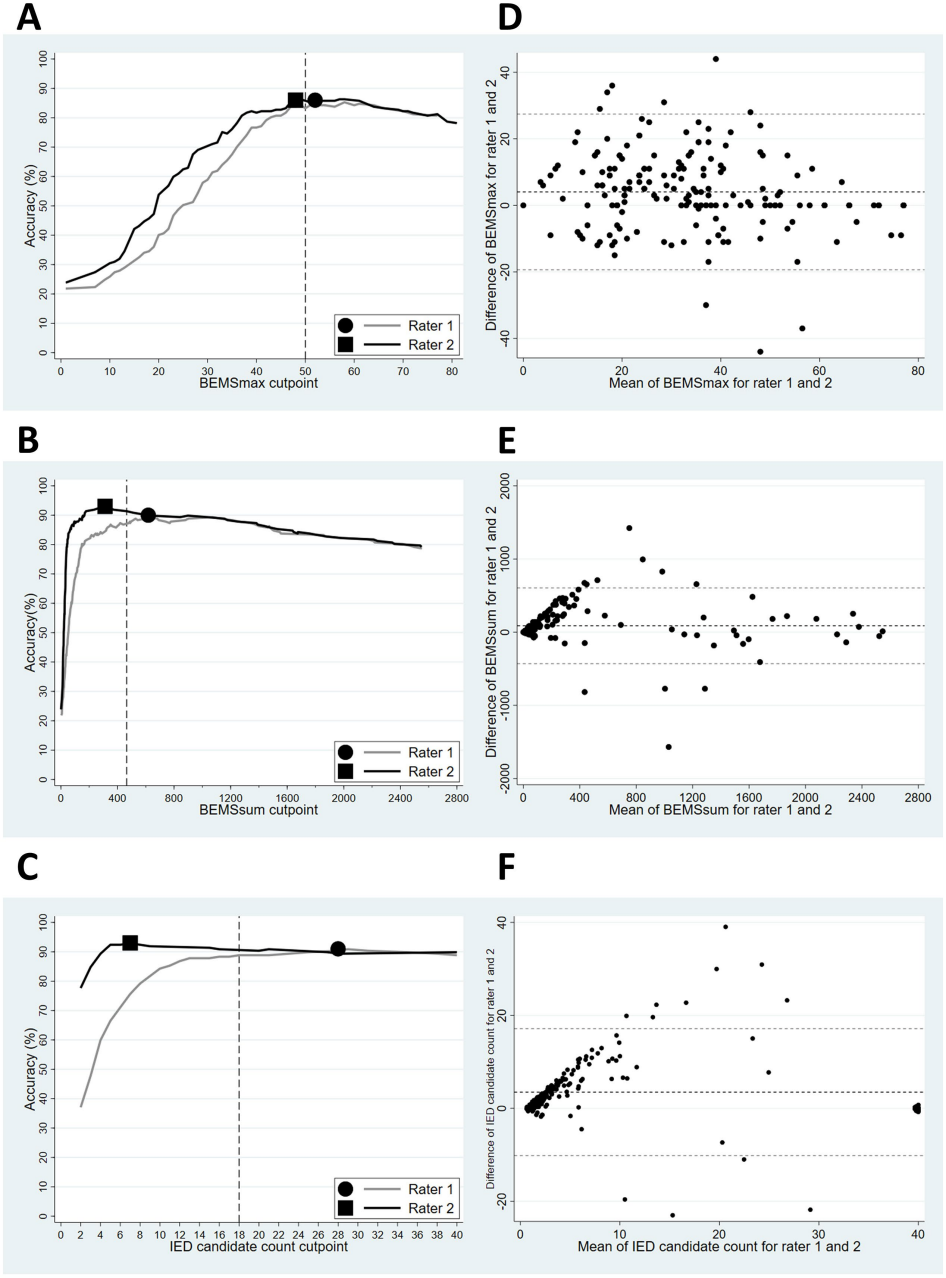
**(A–C)**: Accuracy for BEMS_max_ **(A)**, BEMS_sum_ **(B)** and IED candidate count **(C)**, with the EEG conclusion as outcome for rater 1 and 2 in DS1. The optimal rater specific cut points are indicated by a dot (rater 1) and a square (rater 2). The vertical dashed lines indicate common cut points. **(D–F)**: Bland–Altman plots for BEMS_max_ **(D)**, BEMS_sum_ **(E)**, and IED candidate count **(F)** for rater 1 and 2 in DS1. Mean values are plotted along the *x*-axis, mean differences between rater 1 and 2 along the *y*-axis. BEMS_max_ had the most evenly distributed means between rater 1 and 2, while BEMS_sum_ and IED candidate count had increasing differences between the two raters for higher means.

The accuracy and IRA data for BEMS_max_, BEMS_sum_, and IED candidate count in DS1 are shown in Figure 2. The cut points applied for all three raters were 50 for BEMS_max_, 465 for BEMS_sum_, and 18 for IED candidate count. The IRA was substantial for all diagnostic markers; ICC and Gwet’s AC1 were 0.76 (95% CI = 0.69–0.81) and 0.88 (95% CI = 0.85–0.96) for BEMS_max_, 0.68 (95% CI = 0.59–0.75) and 0.86 (95% CI = 0.80–0.93) for BEMS_sum_, and 0.73 (95% CI = 0.65–0.79) and 0.90 (95% CI = 0.83–0.95) for IED candidate count, respectively. The diagnostic performance when applying the common cut points for the individual raters was as follows: For BEMS_max_, the sensitivity was 64% (95% CI = 48–78), specificity was 89% (95% CI = 83–93), and accuracy was 84% (95% CI = 78–89) for rater 1 and sensitivity was 64% (95% CI = 48–78), specificity was 92% (95% CI = 86–96), and accuracy was 86% (95% CI = 80–90) for rater 2. For BEMS_sum_, the sensitivity was 71% (95% CI = 55–84), specificity was 92% (95% CI = 87–96), and accuracy was 88% (95% CI = 82–92) for rater 1 and the sensitivity was 64% (95% CI = 48–78), specificity was 99% (95% CI = 95–100), and accuracy was 91% (95% CI = 87–95) for rater 2. For IED candidate count, the sensitivity was 64% (95% CI = 48–78), specificity was 96% (95% CI = 92–99), and accuracy was 89% (95% CI = 84–93) for rater 1 and the sensitivity was 60% (95% CI = 43–74), specificity was 99% (95% CI = 95–100), and accuracy was 90% (95% CI = 85–94) for rater 2. The diagnostic classifier with the highest combined accuracy and IRA for both raters was as follows: One IED candidate with BEMS > = 58, two IED candidates with BEMS > = 47, or seven IED candidates with BEMS > = 36. The sensitivity, specificity, and accuracy were 67% (95% CI = 51–80), 92% (95% CI = 86–95), and 86% (95% CI = 81–91) for rater 1 and 62% (95% CI = 46–76), 96% (95% CI = 92–99), and 89% (95% CI = 84–93) for rater 2, respectively. IRA was almost perfect with Gwet’s AC1 = 0.89 (95% CI = 0.83–0.95).

### Diagnostic performance in DS2 and DS3

Diagnostic performance for the various markers in DS1 and DS2 is shown in Table 2. Gwet’s AC1 was >0.89 for all diagnostic markers in DS2 and varied from 0.57 to 0.73 in DS3. The diagnostic classifier had the highest IRA in both datasets with Gwet’s AC1 of 0.96 in DS2 and 0.73 in DS3. The sensitivity for the markers was 60–67% in DS2 and 33–70% in DS3. The specificity was 96–99% in DS2 and 86–91% in DS3. Supplemental Digital Content 1 shows rater-specific performance measures with 95% confidence intervals. The diagnostic performance of our current standard of care, the clinical EEG conclusion, which also served as a reference standard for the diagnostic markers, had a sensitivity of 41% (95% CI = 28–56) and a specificity of 97% (95% CI = 93–99) for the follow-up diagnosis of epilepsy in DS2.

**Table 2:** Mean performance of diagnostic markers between 3 raters when applying the common cut points that were developed in DS1 for rater 1 and 2.

	DS2 (*N* = 187)					DS3 (*N* = 60)		
	Interrater agreement	EEG conclusion		Epilepsy		Interrater agreement	Epilepsy	
	Gwet’s AC1	Sensitivity %	Specificity %	Sensitivity %	Specificity %	Gwet’s AC1	Sensitivity %	Specificity %
BEMSmax*	0.90	60	96	29	95	0.57	70	86
BEMSsum**	0.94	67	98	32	98	0.69	56	94
IED candidate count***	0.93	60	98	29	97	0.70	33	96
Diagnostic classifier****	0.96	60	99	26	97	0.73	63	91
Clinical EEG conclusion				51	96			

Asterisks indicate appliance of common cutpoints. *: BEMSmax > = 50. **: BEMSsum > = 465. ***: IED candidate count > = 18. ****: either 1 IED candidate with BEMS > = 58, 2 with BEMS > = 47 or 7 with BEMS > = 36.

## Discussion

We have shown in a large study of routine scalp EEGs that there is a positive correlation between characteristic epileptiform IED morphology and IED candidate count. EEGs with distinct epileptiform discharges had a high IED candidate count, while EEGs with less characteristic epileptiform activity as defined by BEMS had a lower IED candidate count. Quantified EEG spike morphology (BEMS) and IED candidate count can be combined to classify an EEG as epileptiform with high reliability but with somewhat lower sensitivity than regular visual EEG review.

Interictal epileptiform discharge (IED) candidate count was an important predictor of epileptiform activity in our study. We suggest that the IED candidate count should be added in a future update of the criteria for epileptiform discharges that was proposed by the International Federation of Clinical Neurophysiology (IFCN). IED candidate count is not an inherent property of epileptiform morphology but rather provides a context for its interpretation. We suspect that a higher IED candidate count strengthens the EEG reader’s confidence in spike detection and reduces the likelihood of false positives, a well-known challenge in visual spike detection (23). Background noise can imitate epileptiform activity once or twice but not repetitively. Some physiological sharp transients can occur repeatedly but have recognizable morphology (21). When spotting a definite epileptiform discharge by visual interpretation, less prominent discharges in the same region are more easily included as IEDs. It depends on the signal-to-noise ratio whether one or more discharges are needed to distinguish a spike from background activity. Signal averaging is a well-known method in signal analysis and applies the same principle. Each addition of raw signal to the running average flattens background noise while the signal of interest remains unchanged. While one epileptiform discharge might fulfill only a few IFCN criteria, the average of many discharges meets more criteria, and morphological uncertainties are eliminated.

We have described the IED candidate count in a large dataset where all EEGs had at least one epileptiform or non-epileptiform sharp discharge scored at the time of the clinical EEG report. The average IED candidate count was estimated to be between two and eight per 20-min EEG. IED candidate count is entirely based on visual interpretation of scalp EEG and does not reveal the intracranial or “true” spike count. Some intracranial IEDs are not detected in scalp EEG (24). Their visibility depends on variables, such as source depth, cortical area, and geometry (25).

Our study had a high IRA. To be able to test the reproducibility of our classification model, we divided our internal EEGs into two independent data sets (DS1 and DS2) and used the second data set for validation, also including a third EEG rater. The inter-rater agreement was substantial to almost perfect between the three raters for the diagnostic classifier, demonstrating robustness regarding variability in the selection of IED candidates between the raters. Our assessment of IED candidate morphology is entirely objective by applying the algorithm for the BEMS score, analogous to a subjective visual assessment of epileptiform criteria. The BEMS algorithm is publicly available for use by equipment manufacturers.

The diagnostic classifier was built using a traditional and explainable analytic approach that classified EEGs as epileptiform or non-epileptiform with high reproducibility and specificity but with lower sensitivity than a routine clinical EEG examination. Possible explanations for the limited sensitivity could be that the BEMS score did not capture enough information per IED candidate or that the clinical information available to the clinical interpreter of the EEG contained decisive information to tip the scales. We found a gray area of ambiguous EEGs that had neither infrequent and unconvincing IED candidates nor numerous and highly epileptiform IED candidates. Experienced clinical EEG interpreters can add diagnostic value in such cases.

Future studies of EEG interpretation should focus on difficult borderline EEGs.

There are several limitations to this study. We did not have access or the capacity to analyze clinical and paraclinical patient data that can be thought to influence or explain BEMS and IED candidate count, e.g., what evidence was available to the clinicians that diagnosed the patients with epilepsy, type of epilepsy syndrome, seizure burden, use of anti-seizure medication, imaging data, neurological comorbidities, IED candidate localization, and topography. Individual rater threshold differences were relatively large for IED candidate count and BEMS_sum_ (Figure 2), affecting the diagnostic performance negatively since a common threshold will differ from each of the rater’s optimal cut point. The IED candidate count threshold differences imply that the difficulties when deciding whether an EEG waveform is an IED candidate are comparable to that of IED classification. The low inter-rater threshold difference and healthy Bland–Altman plot for BEMS_max_ (Figure 2) suggest that the IED candidate selection by a human rater combined with automated quantitative BEMS scores reliably identifies the IED candidate with the most typical epileptiform characteristics in an EEG.

We validated the diagnostic performance and IRA of the diagnostic markers by examining an external EEG dataset (DS3) that had different patient characteristics, prevalence of positive outcomes, and reference standards. DS3 consisted of patients who had required long-term video-EEG monitoring (LTM) in their work-up, as opposed to our internal datasets which included routine EEGs from a wide variety of referrers and reasons for requesting an EEG. The pretest IED probability was lower in DS1 and DS2 compared with DS3. The low prevalence of focal IEDs in our internal datasets approximates the actual prevalence in the patient population that is referred to our EEG laboratory for a routine EEG, which can be estimated at 8% from our previous studies (18). Validating the diagnostic markers on DS3 was a “trial by fire” due to the different dataset characteristics outlined above. The optimal cut point for any quantitative diagnostic marker depends on pretest probability, and its application will be more suitable for similar datasets. The gold standard in DS3, the classification of habitual seizures as epileptic or non-epileptic by LTM, is an outcome measure of a higher standard than those in DS1 and DS2, which were focal IEDs as classified by the attending physician and the presence of a follow-up diagnosis of epilepsy in the hospital database records.

## Conclusion

Interictal epileptiform discharge (IED) candidate count is a relevant predictor variable in the classification of EEGs as epileptiform or non-epileptiform. IED candidate count correlated positively with IED candidate morphology. Based on our data, we suggest the following criteria for definite interictal epileptiform activity: either at least one very typical epileptiform discharge (BEMS> = 58), or at least two moderately typical epileptiform discharges (BEMS> = 47), or at least seven less distinct epileptiform discharges (BEMS> = 36).

## Data availability statement

The datasets presented in this article are not readily available because of ethical and privacy restrictions. Requests to access the datasets should be directed to the corresponding author.

## Ethics statement

The studies involving human participants were reviewed and approved by REK vest, Universitetet i Bergen, Det medisinske fakultet, Postboks 7,804, 5,020 Bergen. The reference code for our research project is “2017/1512/REK vest.” Written informed consent from the participants’ legal guardian/next of kin was not required to participate in this study in accordance with the national legislation and the institutional requirements.

## Author contributions

EA drafted the manuscript. EA, NG, and JB were responsible for the study design, methodology, and revising the manuscript. EA, JB, and HO collected data for the internal dataset. SB and MK provided and organized the external dataset. EA and JB performed the statistical analysis. All authors contributed to the article and approved the submitted version.

## Funding

This work was supported by Helse Vest, project number F-10226.

## Conflict of interest

JB, EA and HO are minority shareholders in Holberg EEG AS, the providers of the SCORE EEG software used in this study.

The remaining authors declare that the research was conducted in the absence of any commercial or financial relationships that could be construed as a potential conflict of interest.

## Publisher’s note

All claims expressed in this article are solely those of the authors and do not necessarily represent those of their affiliated organizations, or those of the publisher, the editors and the reviewers. Any product that may be evaluated in this article, or claim that may be made by its manufacturer, is not guaranteed or endorsed by the publisher.
